# Impact of Cardiac Rehabilitation on Functional Capacity and Physical Activity after Coronary Revascularization: A Scientific Review

**DOI:** 10.1155/2020/1236968

**Published:** 2020-03-21

**Authors:** Niramayee V. Prabhu, Arun G. Maiya, Nivedita S. Prabhu

**Affiliations:** Department of Physiotherapy, Manipal College of Health Professions, Manipal Academy of Higher Education, Manipal, Karnataka 576104, India

## Abstract

**Background:**

Coronary revascularization procedures often cause lowered exercise capacity and declining physical activity levels. These outcomes are paramount in predicting morbidity and mortality after these procedures. Cardiac rehabilitation (CR) focuses on incrementing cardiovascular endurance, exercise capacity, muscle strength, levels of physical activity, and quality of life through health education and lifestyle modification in post-coronary revascularization patients.

**Objective:**

To review the impact of cardiac rehabilitation on functional capacity, levels of physical activity, and health related quality of life in patients following coronary revascularization.

**Methods:**

A structured literature search in PubMed, Scopus, CINAHL, and ProQuest for studies focused on CR and its effects on functional capacity, physical activity, and quality of life after coronary revascularization.

**Results:**

A total of 2,010 studies were retrieved. Deduplication and eligibility screening included 190 studies after the application of filters. A sum of 21 studies were considered for this review. Most studies reported that exercise and physical activity programs were centre-based and home-based and via telerehabilitation. Treadmill and cycle ergometry training with callisthenics and flexibility exercises in phase 2 CR exhibited significant improvement in functional capacity and physical activity levels in patients after coronary revascularization procedures.

**Conclusion:**

Although centre-based supervised CR programs do improve functional capacity after coronary revascularization, home-based or telerehabilitation-based CR programs are feasible, improve patient compliance in improving physical activity, and thereby increase functional capacity. Patient education improves levels of physical activity, health related quality of life, and adherence to home- and centre-based CR programs.

## 1. Introduction

Cardiovascular disease (CVD) accounts as a leading cause for global mortality and morbidity under the noncommunicable disease spectrum. The Global Burden of Disease (GBD) framework reports CVD as the most common cause for all deaths from 1990 to 2015 [1]. An estimated 17.9 million deaths (31 percent) were reported by the World Health Organization (WHO) in 2016 exclusively due to CVD [2]. Coronary artery disease (CAD) is the most frequently encountered cardiovascular disease and is associated with a decline in quality of life [2, 3]. Revascularization procedures such as Coronary Artery Bypass Graft (CABG) surgery and Percutaneous Coronary Intervention (PCI) are ideally sought for the management of CAD [4]. These procedures are associated with declining functional capacities due to plausible ebb-flow phases occurring from the surgical stress, hospitalization, and drug related effects further leading to lowered levels of physical activity [5, 6, 7]. However, CABG surgery has been observed to positively influence physical and mental health leading to an improved health related quality of life (HRQoL) after a period of 5 years [8]. A meta-analysis of HRQoL scores showed similar improvement in quality of life with minimal differences between PCI and CABG patients [9]. Alternately, a retrospective cohort observed a decline in HRQoL presenting a strong association between preoperative and postoperative scores under the physical and mental domains [10]. Following these revascularization procedures, issues of lowered aerobic capacity, levels of physical activity, and quality of life necessitated referrals to cardiac rehabilitation programs as an essential constituent for holistic treatment in patients with CVD [5, 7, 10–13].

Cardiac rehabilitation (CR) is a multidisciplinary approach implemented in patients after an adverse cardiac event. It aims at improving the patient's activities of daily living, thereby aiding in early return to work. Education about cardiac disease, behavioural counselling after an adverse event and its prognosis, exercise (aerobic versus resistance, centre-based versus home-based) training, and lifestyle management form essential components of a CR program. A CR program includes rehabilitating patients from the acute (in-hospital) phase to a home-based or community-based setting. Before and after cardiac surgery, the goal of CR is to optimise a patient's functional status and aerobic capacity. Phase 2 CR primarily focuses on testing and improving exercise capacity of the cardiac patient over a period of six weeks after the in-hospital phase (phase 1) [11]. Treatment strategies over time have been modified and various methods of treatment have been incorporated in CR [14].

Frequently focused outcomes during cardiac rehabilitation are based on either performance or quality measures. A recent task force report states five clinical performance and three quality-based measures to implement CR in the centre or community across various encountered challenges. Performance measures were designed as referrals to CR in the inpatient/outpatient settings, exercise training for heart failure (HF) in both inpatient/outpatient settings, and enrollment in CR through an electronic health record or registry. Time taken to CR patient enrollment, adherence to CR (>36 sessions), communications about adherence, enrollment, and outcomes were categorized as quality measures with a need for testing [11].

Formerly cardiac rehabilitation focused on implementation of centre-based rehabilitation programs. However with the advent of time, home-based and e-health programs (telerehabilitation) were designed trying to make CR accessible to those patients who were unable to avail benefits of centre-based programs [14–16]. Benefits of participation in regular CR programs have proven to reduce all-cause mortality by more than 12% after CABG over a duration of 10 years [17]. Although these CR programs do provide significant medical supervision, patient output was reported to be poor at rehabilitation centres after events of myocardial infarctions (MI), coronary artery bypass graft surgeries, and percutaneous coronary intervention [18]. The effects of CR on functional capacity, physical activity, and quality of life have been vastly reported but these are not exclusive to the effects of phase 2 CR.

This review aims at studying cardiac rehabilitation in patients who have undergone coronary revascularization procedures and its impact on their functional capacity, levels of physical activity, and health related quality of life.

## 2. Methods

**Figure 1:**
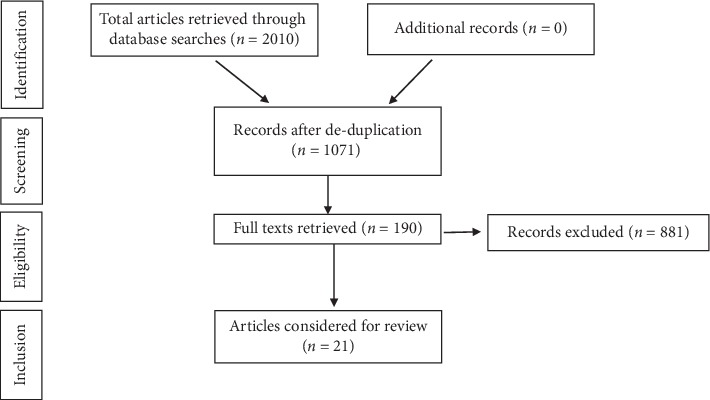
PRISMA flowchart of studies.

Databases accessed included PubMed, ProQuest, Cumulative Index to Nursing and Allied Health Literature (CINAHL), and Scopus. Human studies and English language were the filters applied with a search duration of 10 years (2009–2019). Keywords selected were coronary artery bypass graft surgery, coronary revascularization, percutaneous transluminal coronary angioplasty, percutaneous coronary intervention, cardiac rehabilitation, functional capacity, six-minute walk test, and physical activity. The MeSH terms used were rehabilitation, cardiac rehabilitation, coronary revascularization, and physical activity. Boolean phrases used were AND, OR, NOT. A flow chart of the systematic screening and inclusion of eligible studies has been presented in Figure 1 as applied in Preferred Reporting Items for Systematic Reviews and Meta-Analysis (PRISMA) method.

## 3. Results

A total of 2,010 studies were retrieved after using keywords, Boolean phrases, and filters from the databases (Figure 1). The deduplication process yielded 1071 studies. On further screening for full text availability, 190 studies were obtained. Screening the title, 21 studies were considered relevant for this review and eligible on basis of full text availability. Studies included randomised clinical trials, quasi experimental trials, prospective cohorts, systematic reviews, and meta-analyses. A summary of details of the studies is represented in Table 1.

## 4. Discussion

### 4.1. CR and Functional Capacity

As a major clinical outcome of cardiac rehabilitation, functional capacity has been one of the key factors in secondary prevention of CVD [4]. Coronary revascularization procedures have been shown to negatively affect functional capacity in patients, which may lead to sedentary lifestyle adaptations [38, 39]. Submaximal exercise stress tests, with better applicability, including performance tests such as six-minute walk test (6MWT) are frequently applied for evaluating functional capacity [40]. The 6MWT implemented at the commencement and completion of phase 2 CR [41] is routinely denoted and calculated as peak VO2 uptake (peak oxygen uptake); functional capacity has been observed to have been improved with CR. The programs focused on improving the distance walked, i.e., aerobic capacity of patients as an outcome of CR, thereby improving the functional capacity [11].

Earlier studies focused on centre-based programs for phase 2 rehabilitation in which patients would visit the centre at least 3 to 5 times a week. The rehabilitation program varied from modes of treadmill training and cycle ergometry training. Both treadmill and cycle ergometers have shown to be equally effective during the training periods [19, 41, 42]. Patients would also perform certain home exercises on the remaining days. Flexibility with callisthenics was included as a part of the rehabilitation program. In few studies, programs progressed from 45 minutes to 1 hour per day [21, 25, 27, 36]. These were performed twice daily with home exercises on the days centre-based rehabilitation was not conducted [27, 34].

Modes of continuous moderate exercise and aerobic interval training have been tested in the CABG population under aerobic exercise training programs. The continuous moderate exercise training group were trained on treadmill for 45 minutes at 70% maximal heart rate (HRmax) while the aerobic interval training group walked for 4 minutes at 90% HRmax in 4 sets with rest periods of 3 minutes each at 70% HRmax. It was observed that, for a short-term effect, both groups were equally effective in improving their functional capacities. However, when assessed for long-term effects, interval aerobic training proved to be effective [30].

A new concept of power walking introduced has increased upper limb involvement along with treadmill walking. This proved that power walking was more beneficial than regular treadmill walking in improving functional capacity and exercise capacity. With the advent of research, it was observed that these services had not been fulfilling the needs of patients [28]. Patients did not attend centre-based programs and were hence deprived of the benefits these programs provided. A study identified barriers to CR, which varied with age groups. The younger age group reported time constraints and work load as one of the major barriers to adhere to CR. Being unable to meet time demands for prescribed set of exercises led to ignoring it as a whole [43]. The older age groups were generally not aware of the benefits of cardiac rehabilitation on their overall health or considered that the problems could be self-managed [28, 43].

Home-based programs were introduced to bridge the existential gap between the use of phase 2 cardiac rehabilitation and its utilization. Home-based rehabilitation provides certain exercises that had been prescribed along with aerobic training at the time of discharge from the centre after a brief evaluation of the status of the patient [14, 21, 25, 44]. The focus was on aerobic exercise either on cycle ergometer or walking with callisthenics [14, 25].

Various studies have been conducted to compare effects of centre-based and home-based programs [14, 21, 44]. All studies maintained a follow-up either telephonic or through some messages to modify the program or to be aware about the adherence of the individual. Most of the studies concluded that both programs are equally effective in improving the functional capacity of the individual [12, 14, 21].

As a measure to improve the quality of home-based CR programs, telerehabilitation has been introduced as an additional mode of delivery. Video-assisted programs and mobile-based follow-ups have been utilized to provide an added benefit to the people availing home-based programs. These have been effective to track adherence and quality of exercises performed at home. An improvement in patient compliance and understanding was reported, making them more adherent to these programs [15, 16].

### 4.2. CR and Physical Activity

Physical inactivity is considered to be a strong risk factor for the development of CVD [12]. According to WHO, an adult should fulfil a total of at least 150 minutes moderate physical activity in a week [20]. After coronary revascularization procedures, it has been observed that patients fail to meet this standard requirement of physical activity [31]. Due to difficulties in performing activities after revascularization, patients generally avoid performing a few of their activities of daily living (ADL). Participation restriction is observed due to the fear of symptom aggravation [45]. A lack of knowledge about difference between physical activity and exercise resulted in an overall drop in the levels of PA [32]. In addition, barriers of limited time restricted patient participation in CR programs towards increasing levels of PA and exercise among coronary revascularized patients [28].

Physical activity is considered a core component of cardiac rehabilitation making it essential along with exercise prescription. It became an integral part of the cardiac rehabilitation program to educate patients and caregivers about its significance for the cumulative health of individuals [12]. Studies that focused on physical activity have tried to incorporate these programs with educational and behavioural methods into the home-based cardiac rehabilitation programs [16, 28, 31, 46].

Physical activity programs were decided according to normally performed activities or according to preference of the patient. Furthermore, behavioural counselling sessions were administered, which added a psychological aspect into the program [31, 46].

Assessment methods between studies vary from subjective (questionnaires—disease specific, self-reported, or global PA questionnaires, e.g., Paffenbarger physical activity questionnaire, seven-day recall questionnaire) to objective (use of pedometer or accelerometer for gauging levels of physical activity) [16, 28, 46]. These studies conclude that, after a physical activity program, an overall improvement was observed in the functional status and physical activity levels [16, 31, 43, 46]. In addition, it was observed that adherence rates were higher while assessing its long-term effects [28]. A recent scientific statement on home-based cardiac rehabilitation, which reviewed 23 studies, extensively analysed the core component of exercise training (home-based or centre-based CR) and its effect on the levels of physical activity in patients undergoing rehabilitation [35]. Increased levels of physical activity leading to an upsurge in functional capacity were noted although recommended use of alternate modes of training due to certain barriers (safety, infrastructure) was alternately advocated [47].

Application of accelerometers and pedometers was analysed in different cardiometabolic conditions (e.g., diabetes and cardiac disease) in a systematic review [26], which reported increased moderate to vigorous physical activity (MVPA) levels with either pedometers or accelerometers specifically when face to face consultation with the health professionals was executed. Regular advice and guidance on exercise or physical activity regime to patients were provided with pedometers and accelerometers separately or in combination yielded higher PA levels with a standardized mean difference of 0.30 (accelerometers) and 0.52 (1703 steps using pedometers). Self-monitored PA, however, did not seem to provide a significant increase.

A systematic review reports effect of patient education in cardiac patients, which assessed its role in improving physical activity [23]. Of the 42 studies included, 26 studies reported PA as a primary outcome. Twenty studies reported an improvement in PA levels with patient education, 5 studies reported no improvement, and one study showed a decrement in PA with education.

### 4.3. CR and Quality of Life

Coronary revascularization procedures often lead to impaired quality of lives due to prolonged hospital stays and the impending physiological stress [11]. Quality of life is associated with changes in the aerobic capacity as well as the psychological status of the patient [48]. In studies that have focused on improvement of behavioural methods and those that have worked on increasing the physical activity, these have shown a significant change in the quality of life. These changes have been observed in both exercise and the physical activity groups but significantly in those that have had behavioural associated counselling present as a part of the cardiac rehabilitation program [33, 49]. Training inspiratory muscles at moderate thresholds for twice a week under an inspiratory muscle strengthening (IMT) program proved beneficial in improving the health related quality of life and functional capacity, thereby linking aerobic capacity and quality of life after 12 weeks in patients after coronary artery bypass graft surgery [22]. In recent times with advancing technology, application of telerehabilitation and telemonitoring in cardiac disease patients placed at remote locations would connect them to health professionals at various centres. A program of 12-week cardiac telerehabilitation (REMOTE-CR) proved to be beneficial in improving both health related quality of life and physical activity in cardiac patients after revascularization [29].

### 4.4. Prognostic Implications of CR

The visibility and types of CR programs vary across the globe [50]. Depending upon modes of delivery, methods of patient education, accessibility to CR centres with supervised exercise programs, efficacy of cardiac rehabilitation can be predicted. Astute clinical competency in a multidisciplinary team with evidence-based prescription of exercises is needed for appropriate delivery of CR programs [51]. Additional factors that influence the implications of an effective CR program consist of adherence and completion. Data collected about utilization of medicare benefits to analyse levels of participation, adherence, and completion (>36 sessions) of CR programs revealed higher participation among patients who underwent CABG and PCI after myocardial infraction (MI) when compared to MI alone, with men more than women and age groups of 74 to 85 years. Patients with multiple comorbidities (>5 comorbidities) after cardiac events presented with lower participation [52]. Implementation of CR programs at smaller tertiary hospitals and community centres, awareness about their efficacy, and beneficiaries through medicare would aid in higher participation after coronary revascularization procedures and improve functional parameters like aerobic capacity, physical activity, and quality of life.

### 4.5. Strengths

The link between various parameters and cardiac rehabilitation has been appropriately reported in the above studies. Follow-up methods, various modes of exercise delivery, and outcome assessments have been mentioned.

### 4.6. Limitations

Long term follow-up after cardiac rehabilitation is less discussed in some studies. There have been a dearth of interventional studies in patients after coronary artery bypass graft surgery and percutaneous coronary interventions. Additionally, studies focus on exercise prescribed cardiac rehabilitation when compared to assessment of physical activity as a primary/secondary outcome. Exercises implemented may not be easily available, which could generate a unidirectional bias in a specific population.

## 5. Conclusion

Based on the studies reviewed, we observed that adherence to cardiac rehabilitation programs under different settings (centre-based, home-based, or telerehabilitation) improves functional capacity, physical activity, and health related quality of life after coronary revascularization.

## Figures and Tables

**Table 1:** Summary of reviewed studies.

S. No.	Author/Year	Design	Population	Intervention	Outcome	Results
1	Anderson et al. 2016 [19]	Systematic review and meta-analysis	RCTs (*n* = 63) CHD inclusive of myocardial infarction (*n* = 36), CABG (*n* = 29) and PCI (*n* = 18)	Duration ranged from 3 months to 3 years (maximum in 6–12-month range)	HRQoL (20)	Heterogenicity in data was seen >50% improvement in the scores was seen in exercise-based rehabilitation

2	Blanchard et al. 2010 [20]	Pre-post-test design	*n* = 280 MI: (*n* = 94), PCI: (*n* = 92) CABG: (*n* = 48) other: (*n* = 46)	3-month home-based program	1. Physical activity (Godin leisure time questionnaire) 2. Body composition	Increase in PA was larger in males (*r* = −0.19), more in metabolic group (*r* = −0.16)

3	Deskur-smielecka et al. 2011 [21]	Controlled prospective cohort	*n* = 74 post-PCI CR and ambulation: (*n* = 14) CR: (*n* = 30) control: (*n* = 20)	1-year follow-up, 3-week in-patient, after 6 weeks the CR and ambulation were on a 3–4 times/week ambulatory program	Body composition	Body composition and BP increased in controls significantly (*p* < 0.05, *p* < 0.01) the BP in CR group compared to CR and ambulation also increased (*p* < 0.05)

4	Dos santos et al. 2019 [22]	RCT	Total: (*n* = 24) moderate to high intensity inspiratory muscle training (*n* = 12), resistance/combined training (*n* = 12)	2 sessions/week for 12 weeks	1. Exercise capacity 2. Respiratory muscle strength 3. Inspiratory muscle endurance 4. QoL	There was an overall increase in the oxygen uptake, 6MWT, maximal inspiratory pressure, and QoL

5	De Melo ghisi et al. 2014 [23]	Systematic review and meta-analysis	*n* = 42 studies (26 studies analysed PA as primary outcome)	Patient education and PA levels	Physical activity levels and adherence to exercise after patient education in cardiac patients	Patient education was elementary in improving levels of PA, dietary habits, and smoking cessation

6	Firouzabadi et al. 2014 [24]	RCT	*n* = 70 post-CABG control: (*n* = 35) intervention: (*n* = 35)	24–32 sessions, 3 times/week, aerobic exercise on treadmill or cycle ergometer for intervention group	QoL (SF-36 QoL questionnaire)	After 4 months there was a significant difference between the scores of both groups (*p* < 0.001)

7	Ghashghaei et al. 2012 [25]	RCT	*n* = 32 post-CABG control: (*n* = 15) Rehab: (*n* = 17)	Control-15–20 mins walking 2-3 times/week Rehab-60 mins aerobic training 60–85% HR max, 3 times/week	1. Functional capacity (6MWT) 2. Ejection fraction 3. Blood pressure, heart rate, rate pressure product	A significant change in the outcomes (*p* < 0.001)

8	Hodkinson et al. 2019 [26]	Systematic review and meta-analysis	*n* = 36 studies (accelerometers *n* = 20 and pedometers *n* = 16)	Face to face consultation and accelerometer/pedometer intervention	PA measured short term and medium term using accelerometers and pedometers (8-month follow-up)	Small to medium improvements were observed in PA from complex accelerometers and pedometers interventions

9	Jelinek et al. 2013 [27]	Pre-post-test design	*n* = 38 patients PCI (*n* = 22), CABG (*n* = 16)	3 times/week for 6 weeks at 55–70% VO_2_ peak Borg scale 11–13 consists of aerobic training and strength training	1. Functional capacity (6MWT) 2. Exercise capacity (VO_2_peak) 3. Heart rate variability	In both there was an increase in the VO_2_peak and 6MWD (*p* < 0.001) for HRV changes were seen in CABG (*p*=0.0072) but not in the PCI group

10	Kim et al. 2012 [28]	Pre-post design	Power walking (PW) group (*n* = 16) and usual walking group (*n* = 18)	The 2 groups have aerobic exercise training on treadmill for 50 minutes/session, 3 times/week for 6 weeks at 60–80% of Hr max. For PW group with upper limb activities. The UW group did the same while holding handle and no upper limb activities	Exercise capacity hemodynamic parameters lipid profile	After the 6-week training, PW group showed better effect than the UW group on the exercise capacity and hemodynamic parameters

11	Maddison et al. 2015 [29]	RCT	*n* = 171 mobile rehab: (*n* = 85) usual: (*n* = 86)	Mobile rehab-30 mins for 5 days/week, automated texts and exercise videos usual-exercise in settings 3 days/week	VO2peak physical activity (IPAQ) HRQoL	No difference in VO2max between groups (*p*=0.65) but PA (*p*=0.05) and SF 36 general domain (*p*=0.03) showed significant difference for mobile group

12	Maddison et al. 2019 [30]	Randomised controlled non-inferiority trial	*n* = 162 REMOTE-CR: (*n* = 82) control: (*n* = 80)	REMOTE-CR: Bespoke telerehabilitation: 30–60 mins > 5 days/week at 40–65% HRR	VO2max lipid profile Anthropometry physical activity HRQOL exercise related motivation Blood pressure	REMOTE-CR is cost effective alternative to centre-based CR. PA {(sedentary: week 24: −61.5 (117.8 to −5.3)}, HRQoL {−0.94(−4.96 to 3.08)}

13	Moholdt et al. 2009 [31]	RCT	After CABG aerobic interval training (AIT): (*n* = 33) moderate continuous training (MCT) (*n* = 36)	5 days/week for 4 weeks AIT-Aerobic exercise 4 mins of 4 intervals at 90% HR max MCT-70% HR max for 46 mins. After 4 weeks, home-based for both	1. VO_2_ peak (exercise capacity) 2. MacNew questionnaire for quality of life	At 4 weeks in VO2max AIT and MCT were effective (*p* < 0.001 for both) at 6 months AIT better than MCT (*p* < 0.001)

14	Oerkild et al. 2010 [32]	RCT	*n* = 75 CHD (MI, CABG, PCI) home-based (HB): (*n* = 36) centre-based (CB): (*n* = 39)	HB-30 mins/day, 6 days/week, Borg scale 11–13 CB-60 mins twice a week after 3 months both home-based. Follow-up-3,6 and 12 months	1. 6MWT 2. VO2max 3. Body composition	Both group interventions were found to be equally effective in improving the outcomes (*p* > 0.05)

15	Peterson et al. 2012 [33]	RCT	After PCI-2 groups physical education (PE): (*n* = 118) physical affirmation (PA): (*n* = 124)	12 months duration. PA-physical activity promotion by self-affirmation and positive affect induction. PE-PA education and goal book	Paffenbarger physical activity and exercise Index	PA group 1.7 times more effective to reach goal than PE (*p*=0.007)

16	Reid et al. 2012 [34]	RCT	Total- (*n* = 141) Motivational counselling (MC): (*n* = 69) usual care (UC): (*n* = 72)	12 months more than 30 mins PA moderate to vigorous ≥5 days/week MC-9 motivational sessions by therapist, telephonic follow-up	7-day physical recall questionnaire	It was seen that PA increased more over MC than UC group (*p* < 0.005)

17	Scalvini et al. 2013 [35]	Quasi experimental study	2 groups: Hospital based (*n* = 100) home-based rehabilitation (*n* = 100)	4-week home-based tele-monitoring of vital, exercise program, hospital-supervised exercises. 100 min/day for both	1. Echocardiogram 2. Functional capacity (6MWT)	Equally significant results for the outcomes (*p* < 0.001) both equally effective

18	Thomas et al. 2019 [36]	Scientific statement from AACVPR/AHA/ACC	*n* = 23 studies (RCT) included with home-based CR	The studies included exercise and physical activity based studies. Behavioural strategies were used	HRQoL exercise capacity physical activity	They concluded that HBCR can help in the delivery of CR services to maximum population

19	Yang et al. 2017 [37]	Systematic review and meta-analysis	6 RCTs *n* = 682 participants	3–6 months, total 30–60 mins/day frequency 2-4 times/day	1. Maximum exercise time 2. Exercise tolerance 3. Angina 4. ST segment decline	It was found that there was a significant improvement in all outcomes (*p* < 0.01)

20	Yates et al. 2017	Descriptive comparative design with secondary analysis of two studies	Two groups: (CABG and HF) *n* = 62	PA examined objectively (ActiHeart accelerometer) and subjectively (PA interview)	Percentage of patients meeting the PA guidelines of ≥150 minutes per week	33% of the CABG patients met the criteria of ≥150 minutes/week of PA No patients with HF were able to fulfil the criteria

21	Yu et al. 2004 [38]	RCT	*n* = 269 acute MI: (*n* = 193), PCI: (*n* = 76)	Cardiac rehabilitation and preventive programs (CRPP)-8-week exercise and educational knowledge with aerobic exercise at 65–85% of HRR. Conventional therapy-no exercise, only educational talk about importance of physical activity	QoL- 1. SF-36 QoL questionnaire 2. Symptom questionnaire 3. Time trade-off questionnaire	SF-36: 6 of 8 sections improved till phase 2 significant changes seen in physical role and functioning

MI, myocardial infarction; PCI, percutaneous coronary intervention; CABG, coronary artery bypass graft; HF, heart failure; CHD, coronary heart disease; HRQoL, heath related quality of life; QoL, quality of life, 6MWT, 6 minute walk test; 6MWD, 6 minute walk distance; CR, cardiac rehabilitation; HRV, heart rate variability; HRR, heart rate reserve; BP, blood pressure; PA, physical activity; SF36, Short Form 36; IPAQ, International Physical Activity Questionnaire; RCT, randomised controlled trial; AACVPR, American Association of Cardiovascular and Pulmonary Rehabilitation; AHA, American Heart Association; ACC, American College of Cardiology.

## Abbreviations

CVD: Cardiovascular disease
CAD: Coronary artery disease
CR: Cardiac rehabilitation
HRQoL: Health-related quality of life
WHO: World Health Organization
MeSH: Medical subject headings
MI: Myocardial infarction
PCI: Percutaneous coronary intervention
CABG: Coronary artery bypass graft.

## Additional Points

*Highlights.* (i) Callisthenics, flexibility exercises as adjuvants to aerobic and strength training improve functional capacity after coronary revascularization. (ii) Methods to improve physical activity emphasised frequently in home-based cardiac rehabilitation program. (iii) Patient education plays a significant role in improving physical activity levels during and after cardiac rehabilitation. (iv) Cardiac telerehabilitation under remote supervision enhances patient adherence to exercise programs. (v) Higher patient adherence, completion, and utilization of cardiac rehabilitation programs observed after revascularization procedures when compared to medically managed cardiac events.
